# Host Shutoff in Influenza A Virus: Many Means to an End

**DOI:** 10.3390/v10090475

**Published:** 2018-09-05

**Authors:** Rachel Emily Levene, Marta Maria Gaglia

**Affiliations:** 1 Graduate Program in Molecular Microbiology, Sackler School of Graduate Biomedical Sciences, Tufts University, Boston, MA 02111, USA; Rachel.Levene@tufts.edu; 2 Department of Molecular Biology and Microbiology, Tufts University School of Medicine, 136 Harrison Ave, Boston, MA 02111, USA

**Keywords:** host shutoff, PA-X, NS1, RNA-directed RNA polymerase, immune evasion, influenza

## Abstract

Influenza A virus carries few of its own proteins, but uses them effectively to take control of the infected cells and avoid immune responses. Over the years, host shutoff, the widespread down-regulation of host gene expression, has emerged as a key process that contributes to cellular takeover in infected cells. Interestingly, multiple mechanisms of host shutoff have been described in influenza A virus, involving changes in translation, RNA synthesis and stability. Several viral proteins, notably the non-structural protein NS1, the RNA-dependent RNA polymerase and the endoribonuclease PA-X have been implicated in host shutoff. This multitude of host shutoff mechanisms indicates that host shutoff is an important component of the influenza A virus replication cycle. Here we review the various mechanisms of host shutoff in influenza A virus and the evidence that they contribute to immune evasion and/or viral replication. We also discuss what the purpose of having multiple mechanisms may be.

## 1. Introduction

100 years after the devastating 1918 Spanish flu epidemic, influenza A virus infections remain a global problem, with only partially effective vaccines and therapeutics available for combating them. Also, many aspects of influenza A virus infection at both the cellular and organismal level remain unclear. Several factors contribute to the complexity of influenza A biology. The remarkable ability of this virus to remodel host processes with its very limited set of 12–17 proteins speaks to the inherent multi-functionality of the influenza A proteins. In addition, the rapidly changing genome and the multitude of hosts that this virus infects means that different strains may behave differently and/or be better adapted to the molecular and organismal biology of different hosts. In this review, we will focus on the process of host shutoff, the global reduction of host gene expression by influenza A virus, as an example of a crucial process that embodies the complexity of influenza biology.

**Figure 1:**
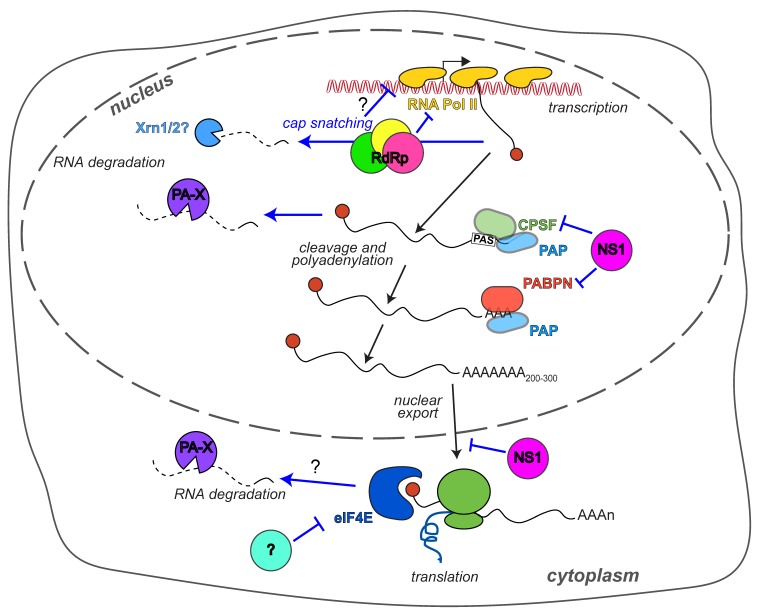
Summary of influenza A virus host shutoff mechanisms. The RNA-dependent RNA polymerase (RdRp) inhibits RNA polymerase II (RNA Pol II) transcription by triggering Pol II degradation. Moreover, because of its cap-snatching activity, the RdRp may promote degradation of nascent RNAs by host exonucleases like Xrn1 and Xrn 2, although the contribution of this degradation to host shutoff is unclear. The process of cap snatching may also contribute to reducing Pol II transcription by reducing Pol II loading on genes. NS1 inhibits 3′ mRNA processing through interactions with the cleavage and polyadenylation complex (CPSF), which recognizes the polyadenylation signal (PAS). Also, interactions between NS1 and the nuclear poly(A) binding protein (PABPN) inhibit elongation of the poly(A) tail by poly(A) polymerase (PAP). NS1 also inhibits RNA export from the nucleus. The RNase PA-X degrades host RNAs in the nucleus and possibly also in the cytoplasm. Unknown factors reduce activation of eIF4E and translation of host proteins.

Reorganizing the host gene expression profile is a powerful way for viruses to overhaul the cell’s biology and promote their own replication. It has long been known that influenza A virus-infected cells display the markings of host shutoff. Early metabolic labeling with radioactive amino acids and nucleotides showed reduced synthesis of host proteins and robust accumulation of viral proteins [1]. They also revealed that both the synthesis and the half-life of host mRNAs are reduced during infection [2,3,4]. These original observations have more recently been expanded using high-throughput RNA sequencing and ribosome profiling to simultaneously measure mRNA levels and translation rates [5]. The results of this approach confirm a general reduction in host RNA levels during influenza A infection that is matched by a reduction in host protein translation [5]. Since the earlier 1970–1990 studies, a plethora of mechanisms have been described that underlie host shutoff and impinge on different aspects of RNA and protein expression. These mechanisms involve at least six viral proteins: The trimeric RNA-directed RNA polymerase complex (RdRp), the non-structural protein NS1, and PA-X, a protein that is generated through a non-canonical translational mechanism and was thus only discovered in 2012 (Figure 1). The purpose of such varied mechanisms of host shutoff in viral replication and immune evasion, their strain and species specificity, and how these mechanisms interact with each other are all questions that are under active investigation. In this review we will detail the state of the research on two questions regarding gene expression changes in influenza A infected cells: What are the mechanisms of host shutoff, and what is the purpose of such pervasive changes in host gene expression. We will then discuss why such an extensive repertoire of tools to control gene expression may exist in such a small virus.

## 2. How Does Influenza A Virus Reduce Host Gene Expression?

### 2.1. PA-X and RNA Degradation

A classical modality of host shutoff is the widespread degradation of host mRNAs. The most well known viral factors that mediate this type of host shutoff are vhs from herpes simplex viruses [6], and the SOX protein from Kaposi’s sarcoma-associated herpesvirus [7]. Early studies showed that influenza A virus also uses this modality of host shutoff, because the degradation of host RNAs is accelerated in influenza A virus-infected cells [3]. However, for many years the RNA destabilization was considered a by-product of cap snatching, a required step in the synthesis of viral mRNAs. Cap snatching refers to the cleavage of host RNA polymerase II (Pol II) transcripts by the influenza RdRp ~11–12 nt from the 5′ end of the transcript, which is modified by a 7-methyl guanosine “cap” [8,9]. These capped oligomers are then used to prime viral mRNA transcription [8,9]. The polymerase acidic (PA) subunit of the RdRp is responsible for the cleavage [10,11]. Cap snatching is necessary because the 5′ cap modification is required to protect mRNAs from 5′–3′ exonucleolytic degradation and to bind factors necessary for mRNA translation in eukaryotic cells. Unlike the RdRp of non-segmented negative strand viruses, the influenza RdRp does not have the enzymatic activity necessary to synthesize the 5′ cap modification. Cap snatching leaves the 5′ end of the host RNAs uncapped, which means that they are now susceptible to degradation, which could in principle explain the destabilization of host transcripts (Figure 1). However, in 2012 Jagger et al. reported the surprising finding that a separate collinear protein encoded from the same genome segment as PA, which they termed PA-X, was responsible for host RNA destabilization (Figure 1) [12]. PA and PA-X share the same 191 amino acid (aa) N terminus, but have different C-terminal domains. The C terminus of PA-X, which is often called the X-ORF, is synthesized using programmed +1 ribosomal frameshifting [12,13]. During translation, the ribosome stalls at a specific decanucleotide sequence in the PA mRNA, a U-rich stretch followed by a rare codon (UCC UUU CGU C) [13]. Forward movement to resolve the stall results in a shift in the codon reading frame, thus generating the X-ORF [13]. Based on in vitro translation assays, this frameshifting event is estimated to occur in only a small fraction of the translation runs, which means that the levels of PA-X are fairly low [12]. Importantly, the shared N-terminal domain of PA and PA-X has ribonuclease (RNase) activity, with a PD-(D/E)-XK fold typical of DNA- and RNA-degrading enzymes [10,11,14,15,16]. The presence of this domain led to the immediate realization that PA-X may act as a host shutoff RNase like vhs and SOX. Importantly, unlike some of the other more recently discovered influenza A virus proteins, PA-X is produced by all influenza A strains [17]. This conservation was revealed by an analysis of 10,164 sequences of influenza A virus isolates, which found that the frameshifting sequence is highly conserved [17]. Moreover, there is a consistent reduction in synonymous diversity in the PA reading frame across influenza A isolates, due to the need to maintain the coding sequence of the +1 X-ORF reading frame [17]. The sequence analysis also revealed that two main variants of PA-X exist: A more common 252-amino acid (aa) variant (191-aa N-terminal RNase domain and 61-aa C terminus) and a 232-aa variant (191-aa N terminal RNase domain and 41-aa C terminus), mainly found in swine and canine strains and in the 2009 pandemic H1N1 strain (henceforth pH1N1) [12,17].

Jagger et al. generated 1918 H1N1 chimeric viruses that produce reduced levels of PA-X because of mutations in the frameshifting sequence, and demonstrated that PA-X-deficient viruses display less efficient host shutoff, as indicated by metabolic labeling assays [12]. In addition, expression of the PA/PA-X mRNA reduces the level of a co-transfected reporter, but this effect is abolished by mutating the frameshifting site, indicating that the RNA degradation is due to PA-X, not PA [12,18]. In the past few years, studies from several other laboratories including our own, have confirmed and extended these results in many other influenza A strains [18,19,20,21,22,23]. Particularly, the results from metabolic labeling and our results with RT-qPCR indicate that PA-X down-regulates a wide range of host RNAs [12,22,24]. In some aspects of its activity, PA-X appears to function similarly to vhs, SOX and the SARS coronavirus host shutoff protein nsp1. All of these proteins preferentially destabilize RNAs that are synthesized by the cellular Pol II [22,25], which transcribes all mRNAs, most long non-coding RNAs and a subset of short non-coding RNAs in the cell. Moreover, the activity of host exonucleases appears to be required to complete RNA degradation after PA-X has fragmented RNAs, also similarly to other host shutoff RNases [22,25]. Lastly, the RNA degrading activity of PA-X results in relocalization of the cytoplasmic poly(A) binding protein to the nucleus and reduction in stress granule formation [26], also similarly to herpesviral host shutoff RNases [27,28]. While the experiments that established these molecular features of PA-X were carried out mostly with the A/Puerto Rico/8/1934 H1N1 (PR8) variant of PA-X, they are consistent with results with variants from other influenza A strains, particularly 1918 H1N1, pH1N1 and H5N1 [12,18,20,21,29]. However, there are unique key aspects of the activity of PA-X. While vhs associates with target RNAs via translation initiation factors [30,31] and SARS nsp1 requires active translation to degrade RNAs [25], PA-X is localized and acts predominantly in the nucleus, at least based on studies with PA-X from PR8 and A/California/4/2009 pH1N1 (henceforth Cal pH1N1) [22,29]. Moreover, it can clearly degrade RNAs that are not translated, such as endogenous long non-coding RNAs, as long as they are transcribed by Pol II [22]. Conversely, translation itself is not enough to direct degradation by PA-X, as this protein does not down-regulate a translated mRNA that does not undergo canonical 3′ end processing [22]. These observations have led us to propose a model whereby PA-X interacts with cellular proteins involved in RNA transcription or processing to associate with its targets, while the other RNases access targets based on translation [25]. The identity of these cellular cofactors remains unknown. It is important to note that one of the consequences of the coupling between RNA degradation by PA-X and Pol II transcription is that viral mRNAs and genomic vRNAs are automatically spared from PA-X activity, as they are transcribed and processed by the viral RdRp [22]. Whether there are additional facets to the specificity of PA-X remains an open question, but in general it appears its effects are widespread, as expected for a host shutoff protein.

While it is clear that the RNase activity of PA-X resides in the N-terminal domain, the role of the unique C-terminal X-ORF in host shutoff is less clear-cut. Multiple groups including our own have shown that removing the X-ORF abolishes the activity of PA-X in cell-based assays in various strains, including PR8, A/WSN/1933 H1N1 (henceforth WSN) and Cal pH1N1 [19,22,29]. In contrast, the PA/PA-X N-terminal domain has RNase activity in vitro [10,11,15,16]. Moreover, almost all influenza A strains have stop codons in the PA-X reading frame after aa 232 and 252, even though there are other earlier positions where a stop codon would not alter the PA coding sequence [17]. This result suggests that shorter X-ORFs are non-functional and thus selected against during evolution and points to a key role of the X-ORF in PA-X biology. Experiments with truncated version of PA-X (from WSN and Cal strains) suggest that the first 15 amino acids of the X-ORFs are sufficient for full activity, while shorter truncations impair host shutoff (Table 1) [19,29]. We have also obtained similar results with the PR8 variant of PA-X (unpublished data). In Jagger et al., 1918 H1N1 viruses carrying a truncated PA-X with a 16-aa X-ORF caused an intermediate phenotype in terms of immune activation [12]. We note that these experimental results seem at odds with the sequence analysis [17], which suggests that there is an evolutionary pressure to retain a full-length X-ORF. Further information on the precise function of the X-ORF and its interactions with cellular factors and RNAs may be needed to resolve this inconsistency. One well-established role for the X-ORF is its function as a non-canonical nuclear localization signal [22,29]. PA-X accumulates in the nucleus, whereas the PA-X RNase domain expressed in isolation does not [22,29]. Nuclear localization appears to be required for PA-X activity, because mutations that prevent nuclear localization also reduce or abolish activity in PR8, WSN and Cal variants of the protein [19,22,29,32]. The nuclear localization activity is conferred by six basic amino acids (R or K) within the first 15-aa of the X-ORF (Table 1) [22,29], although mutations in other parts of PA-X have recently been reported to also affect nuclear localization (Table 1) [32]. Mutation of the K/R X-ORF residues to alanine prevents nuclear localization and abolishes the shutoff activity of PA-X [19,22,29]. However, Hayashi et al. showed that fusing the RNase domain of PA-X from Cal pH1N1 to a classical SV40 nuclear localization signal only partially restores host shutoff activity [29]. This result suggests that the X-ORF has additional functions beyond nuclear localization. Moreover, PA-X with a 9-aa X-ORF is nuclear localized, but has reduced activity, again pointing to additional roles of the X-ORF in host shutoff activity [29]. Bavagnoli et al. showed that in vitro WSN PA and PA-X have greater RNase activity than the WSN PA/PA-X N-terminus alone, but did not distinguish whether this is due to increased RNA binding, better protein stability or changes in enzymatic activity [16]. One possibility is that the X-ORF is responsible for interactions between PA-X and cellular cofactors that guide its association with RNA.

It is also unclear what the significance of the two X-ORF variants (61 aa vs. 41 aa) is, and whether they have differential activity. WSN PA-X has a 61-aa X-ORF, while Cal PA-X has a 41-aa X-ORF, but both of these variants retain full host shutoff activity in reporter experiments when their X-ORF is truncated to 15 aa [19,29]. Nonetheless, Bavagnoli et al. observed higher activity of the longer wild-type isoform of WSN PA-X in vitro, compared to a truncated 41-aa X-ORF version [16]. Another study altered the length of the X-ORF in PA-X from pH1N1, H5N1, and H9N2, thus comparing the activity of 61-aa and 41-aa variants with similar sequences, and consistently showed greater activity of the longer variants in down-regulating a co-transfected GFP reporter [33]. These authors also reported that expressing the C-terminal 20 amino acids of the longer PA-X isoform alone could decrease gene expression, in the absence of the RNase domain [33]. However, this result is not consistent with the fact that mutating the RNase catalytic residues D108 and K134 of PA-X disables its host shutoff activity in every strain tested (for example [12,18,22]). A comparison of PA-X variants from H3N8 isolates from horses and dogs by the Parrish group also found that the longer 252-aa equine PA-X isoforms have slightly higher host shutoff activity in reporter assays than the shorter 232-aa canine isoforms [34]. Also, shortening the X-ORF of the equine PA-X reduced its activity [34]. However, the converse experiment elongating the X-ORF of the canine PA-X did not only increase PA-X activity unless additional mutations were also introduced (Table 1) [34]. This result demonstrates that it is hard to compare 61-aa and 41-aa variants, because they naturally occur in distinct strains, which may also carry other changes in the PA-X sequences that can affect activity and/or compensate for the effect of the X-ORF truncation. Indeed, the 252-aa WSN PA-X has lower host shutoff activity than 232-aa Cal PA-X [18]. It will be interesting to examine the two X-ORFs again when the mechanism of action and cellular interaction partners of PA-X become clearer, so that more precise experiments can be designed. The potential in vivo importance of the X-ORF length was underscored by a study of PA-X in swine strains [35]. Xu et al. highlighted how the X-ORF has become truncated over time in swine influenza isolates, and showed that the 41-aa variant is better at supporting viral replication and transmission in pigs and swine cells [35]. Therefore the two isoforms may simply work more efficiently and be optimized for different hosts.

Because the discovery of PA-X is relatively recent, many aspects of its biology and mechanism of action remain unknown, such as its potential cofactors and regulation of its activity by signaling events. A recent study by Oishi et al. described the first post-translational modification of PA-X, an acetylation at the N-terminal M-E residues of the protein by the host acetylase NatB [36]. This acetylation was present on PA-X isoforms from WSN, Cal (pH1N1), H3N2, H5N1, and H7N9, and stimulates host shutoff activity. However, it is unclear whether the modification is constitutive or regulated, and how it promotes RNA destabilization by PA-X [36]. In general, further mechanistic studies are necessary to define the activity of PA-X and its variations across influenza A strains.

### 2.2. The RdRp and RNA Transcription

While the discovery of PA-X has established that host mRNA degradation during influenza infection is a separate function from cap snatching, cap snatching may still contribute to host shutoff by altering host RNA transcription. Cap snatching requires direct binding between the RdRp and host Pol II [37], because the influenza RdRp acts on nascent RNAs [38] rather than fully processed ones like that of other RNA viruses, for example Bunyaviruses [39]. Indeed, active cellular transcription is needed for influenza A virus replication [40]. The interaction between the Pol II and the RdRp has consequences for Pol II activity (Figure 1). The association inhibits elongation by Pol II, which reduces the amount of cellular RNA transcribed [41,42]. Several forms of Pol II exist in the cells with differential phosphorylation of the heptapeptide repeats Tyr^1^-Ser^2^-Pro^3^-Thr^4^-Ser^5^-Pro^6^-Ser^7^ that comprise the C-terminal domain of the large subunit of Pol II. The heptapeptide phosphorylation follows a stereotypical pattern during transcription [43]. The unphosphorylated form is a pre-initiation form, while Ser5 is phosphorylated upon transcriptional initiation and Ser2 when Pol II is released from the promoter for elongation [43]. Interestingly, the influenza RdRp specifically interacts with the Ser5-phosphorylated form, which is the initiation form, but not with Ser2-phosphorylated Pol II, the elongation form [37]. Furthermore, influenza infection, presumably through the association of the RdRp with Pol II, depletes Pol II from the body but not the promoter of the genes [41]. The reduction in Pol II loading on the host genome was recently explored more in depth by Bauer et al. [42] using mammalian nascent elongating transcript sequencing (mNET-seq), a high-throughput sequencing-based technique that reveals where Pol II is actively transcribing along the genome. mNET-seq showed that cells infected with several influenza A strains (WSN, PR8, A/Udorn/307/1972 H3N2 (henceforth Udorn) and an influenza B strain) display signs of transcriptional dysregulation [42]. Transcription elongation is clearly globally inhibited, as polymerase occupancy in the body of genes is reduced [42]. In addition, the same low level of polymerase occupancy continues after the 3′ transcription termination and polyadenylation sites, indicating that transcription termination is also impaired [42]. While expression of the influenza non-structural protein NS1 can inhibit transcription termination, as discussed more extensively below and shown by Bauer et al. [42], the transcription termination defect was still seen in cells infected with NS1-deficient Udorn or WSN [42]. Given the direct interaction between RdRp and Pol II, it is highly likely that the transcriptional dysregulation is directly triggered by the RdRp. Cap snatching could induce Pol II release from the gene body through the generation of uncapped RNAs that can be attacked by the cellular exonuclease Xrn2. According to one of the current models of termination, the torpedo model, transcription termination is triggered by degradation of uncapped fragments of RNAs by Xrn2 and subsequent interactions between Xrn2 and Pol II [44]. These fragments are normally generated after recognition of the 3′ polyadenylation sequence and cleavage of the nascent RNA [44]. Thus, it is possible that the uncapped fragments generated during cap snatching may be interpreted by the cellular enzymes as post 3′-end cleavage fragments, and that the termination process may be engaged early. 

Another possibility is that reduced Pol II loading on the genome is connected to the degradation of cellular Pol II, an RdRp-dependent event that starts a few hours after infection in multiple influenza A strains, including A/Victoria/3/1975, WSN, and Cal (but not attenuated strains such as PR8 and the cold adapted A/Ann Arbor/6/60) [45,46,47]. Pol II degradation is mediated by the ubiquitin-proteasome system [47]. Moreover, expression of the complete RdRp alone (but not of individual subunits) also induces Pol II degradation [45,47]. A reduction in host transcription occurs at the same time as Pol II degradation, and was detected via the reduced expression of co-transfected reporters [47] and nuclear run on assays [45]. However, it remains formally possible that these gene expression changes are a consequence of Pol II removal from the gene bodies rather than Pol II degradation, or that degradation is secondary to reduced loading. Interestingly, interactions between the RdRp and Pol II are not sufficient for Pol II degradation, because the RdRp of the lab-adapted strain PR8 binds Pol II efficiently, but does not trigger its degradation [46]. The identity of residue 504 in the PB2 subunit of the RdRp and the C-terminal residue 550 of the PA subunit appear to determine whether the RdRp can induce Pol II degradation (Table 1) [48]. Also, the degradation of Pol II starts several hours after infection [45,47]. This temporal delay is consistent with the idea that ongoing cellular transcription is required for viral mRNA transcription, but not viral genome replication. The degradation may thus start once the virus has started shifting from transcription to genome replication [45,47].

### 2.3. NS1 and RNA Processing

The first described host shutoff factor in influenza A virus was the crucial influenza A virus immune regulator NS1. Early studies showed that the NS1 gene is required for viral replication only in interferon (IFN)-competent cells and mice [49]. This result established that the principal function of NS1 is to reduce activation of early type I IFN-mediated responses to infection. Over the years it has become clear that this important function is mediated by several different molecular mechanisms (reviewed in [50,51]), which surprisingly are not shared by all influenza A variants of NS1 [52,53,54,55,56,57]. One of the mechanisms by which many (but not all) variants of NS1 regulate type I IFN is host shutoff, which is accomplished through the inhibition of host mRNA 3′ end processing (Figure 1) [58]. This inhibition is mediated by interactions between the effector domain of NS1 and one of the components of the cellular cleavage and polyadenylation factor complex, CPSF30 (Figure 1) [58]. The CPSF complex recognizes the polyadenylation signals at the 3′ end of mRNAs during transcription, cleaves the RNA and recruits poly(A) polymerase to add the poly(A) tail [59]. The 3′ poly(A) tail of eukaryotic mRNAs, like the 5′ cap, is required for RNA stability, export from the nucleus and translation. NS1 blocks cleavage and polyadenylation of the RNAs, ultimately preventing protein expression. Like the PA-X shutoff activity, NS1 shutoff has no effect on the viral mRNAs, because their poly(A) tails are generated via the stuttering of the RdRp rather than through cleavage and polyadenylation of the RNA [60]. Functional, structural and sequence analysis studies have identified the binding site for CPSF30 on NS1, which is highly conserved among human isolates [61,62,63]. The residues at position 103 and 106 in particular appear to determine whether the NS1 protein can interact with CPSF30 and block polyadenylation (Table 2) [54,61,62]. Generally, NS1 variants with phenylalanine at aa 103 and methionine at aa 106 bind CPSF30 efficiently, while NS1 variants with other amino acids at these positions do not. Indeed, mutating residues 103 and 106 can turn a non-CPS30 binding NS1 into a CPSF30-binding variant [54,64,65]. There are some exceptions, as there are a few NS1s that do not bind CPSF30 despite having F103/M106, such as NS1s from the original pH1N1 strains and circulating canine H3N8 strains [52,66]. A single amino acid change at position 186 is responsible for the lack of CPSF30 binding in the canine H3N8 NS1 [66], which is located in a site previously shown by the Krug lab to be important for CPSF30 binding, aa 184–188 (Table 2) [63,67]. In Cal pH1N1, CPSF30 binding can be experimentally restored by mutating three other residues (R108K, E125D, G189D, Table 2) located at the NS1-CPSF30 interface [52,66,68]. Interestingly, these residues differ between NS1 sequences of swine origin (like that of pH1N1) and “human-like” viruses, and may thus contribute to human adaptation of swine viruses through acquisition of CPSF30 binding [52]. Consistent with this idea, the NS1 of pH1N1 strains currently circulating in human populations have naturally acquired the ability to bind CPSF30 through changes in six amino acids (Table 2), one of which is an E125D mutation [68]. In addition, several other residues have been reported to modulate CPSF30 binding and host shutoff based on analysis of circulating human and veterinary strains and viral mutants (Table 2) [61,69,70,71,72].

Experiments with mutants that can and cannot bind CPSF30 have demonstrated that the NS1-mediated polyadenylation block can potently reduce the expression of type I interferons [67]. Also, transfection of CPSF30-binding NS1 variants down-regulates the expression of unrelated co-transfected reporters [52,53,54,55,56,57]. At present, no dataset is available that addresses the transcriptome-wide effects of NS1 on host mRNA polyadenylation, so it is unclear how global these effects are. Overexpression of NS1 causes a global transcription termination defect based on mNET-Seq data, similarly to influenza A virus infection [42]. Moreover, this defect is analogous to siRNA knock-down of CPSF30, as expected from the NS1-CPSF30 interaction [42]. Despite these results, Bauer et al. found that the termination defect in infected cells is not due to NS1 activity, because it is still seen in cells infected with viruses carrying NS1 deletions or mutations [42]. An alternative approach to understanding the targets and the effect of NS1 on cellular genes will be to use 3′-end and poly(A) tail targeted high-throughput sequencing methods. This approach would specifically interrogate changes in polyadenylation and isolate the effect of NS1 from those of other host shutoff factors. 

A complication in studying NS1 is the multitude of other functions that have been reported for this protein. NS1 can also directly interact with several components of type I IFN signaling (reviewed in [51]) and inhibit the inflammasome [76,77]. It also has additional functions in the regulation of host gene expression that are have not been as extensively characterized as the inhibition of CPSF activity. Udorn NS1 was shown to interact with the nuclear poly(A) binding protein PABPN (or PABII) [78]. This interaction reduces the elongation of the poly(A) tail beyond 10 residues, a process that requires PABPN stimulation of the poly(A) polymerase [78]. The inhibition of poly(A) tail elongation could have additional effects on RNA processing and stability, perhaps as an additional level of regulation for RNAs that are still cleaved in the presence of NS1 [78]. The Fontoura lab also described a separate function of NS1 in blocking export of host RNAs from the nucleus through interactions with nuclear export complex proteins, which they characterized in the WSN strain (Figure 1) [79,80]. Positive effects of H5N1, H5N2, and PR8 NS1 on global translation have been reported [53,56,81], as well as direct interactions of PR8 NS1 with the ribosomes and the RNA regulator Staufen [81,82]. While these additional functions are only partially characterized, they may also be strain specific, like the CPSF30 binding, further complicating the analysis of NS1 function [53]. It will be interesting to dissect these differential components of NS1 activity, in addition to the general role of NS1 in gene expression control.

### 2.4. Translation

Perhaps the most classical mechanism of host shutoff is the shutoff of cellular translation. This modality is exemplified by the poliovirus protease cleavage of translation initiation factors, which are required for binding of cap-dependent translation of host mRNAs, but dispensable for poliovirus translation [83]. Early studies suggested that a similar type of shutoff may occur during influenza infection, and that there is selective translation of mRNAs bearing the 5′ untranslated region (UTR) of influenza A virus mRNAs during infection [84,85,86]. In these studies, a reporter fused to the 5′ UTR of segment 5 (NP segment) from the WSN strain is translated in WSN-infected strains, while a reporter with a control 5′ UTR is not [85]. Several follow up studies have provided evidence that influenza mRNA translation has divergent requirements for translation initiation factors compared to cellular mRNA translation. In particular the Nieto lab has shown that influenza A virus proteins from PR8 and A/Victoria/3/1975 H3N2 are robustly translated in cells with reduced levels or activity of the cap-binding protein eIF4E, while cellular protein translation is severely reduced in the same cells [87]. This finding is consistent with previous observations that eIF4E activity is reduced in WSN-infected cells, because of reduced eIF4E phosphorylation (Figure 1) [88]. In contrast, other translation initiation factors, particularly eIF4GI and eIF4A, are required for influenza A protein translation (at least for the A/Victoria/3/1975 H3N2 strain, [89]), even though eIF4GI phosphorylation is also altered in influenza A infected cells [88]. The low eIF4E dependence of influenza A mRNA translation is not due to inherent properties of the RNA, but likely to the activity of viral or host proteins, because it is not observed in in vitro assays [90]. Transcription by RdRp is required to make translation largely eIF4E-independent, perhaps through direct recruitment of the eIF4GI initiation factor [90]. Moreover, the influenza NS1 proteins from WSN, A/Victoria/3/1975 H3N2, and PR8 have been proposed to promote selective translation of viral mRNAs [86,91,92], although other studies suggest NS1 has a more global effect on translation [53,81]. The translational effect of NS1 could be mediated through interactions with eIF4GI and the cytoplasmic poly(A) binding protein, which is also required for eukaryotic mRNA translation [93,94,95]. However, viruses lacking NS1 can replicate in cells that do not induce type I IFN like Vero cells [49], suggesting this translational role is not a requirement for influenza A replication. While these data point to mechanisms for selective translation of viral vs. cellular mRNAs, the centrality of translational control to host shutoff in influenza A virus has recently been called into question by a global ribosome profiling-based study of cells infected with PR8 [5]. In this study, there was no difference in the translation efficiency of viral vs. cellular mRNAs, and the main source of host shutoff appeared to be a substantial reduction in host RNAs [5]. However, many of the previous studies were done with influenza A strains other than PR8 (A/Victoria/3/1975 H3N2 or WSN), and it is possible that translation-based host shutoff is a strain-specific effect. However, the ribosome profiling results suggest that preferential translation of viral mRNAs is not a universal mechanism of gene regulation among influenza strains.

## 3. Why Does Influenza A Virus Reduce Host Gene Expression?

### 3.1. Host Shutoff as a Mechanism of Immune Evasion

The most commonly proposed function of host shutoff is to reduce induction of immune-related genes. In this model, by preventing general host gene expression, host shutoff also reduces the induction of innate immune chemokines and cytokines, in particular the anti-viral type I and III IFNs, and/or of IFN-stimulated genes (ISGs). This process would therefore block the initial cell-intrinsic protective responses, as well as the recruitment of circulating immune cells like macrophages, neutrophils, B and T cells. For most of the described host shutoff proteins, experimental evidence exists that this is indeed the purpose or at least one of the purposes of host shutoff.

Host shutoff via the degradation of Pol II (Section 2.2) has not conclusively been shown to affect immune responses. However, mutations in the PA and PB2 residues that allow RdRp to trigger Pol II degradation (Table 1) affect the virulence of influenza A strains. A pH1N1 virus with mutations that prevent Pol II degradation is remarkably less virulent than its wild-type counterpart [48]. Since the mutant replicates to similar titers as wild-type in MDCK cells and in mouse lungs, this is not simply due to altered polymerase activity [48]. Moreover, while the PR8 strain does not normally trigger Pol II degradation [46], a more virulent PR8 mutant isolated after passaging in Mx1 +/+ mice [96] carries changes at positions 504 of PB2 and 550 of PA that restore the Pol II degradation function of the RdRp [48,97]. While HA and NA mutations influence the pathogenicity of the high-virulence PR8 isolates [96], the PA and PB2 mutations also contribute to the higher virulence [97].

As mentioned before, NS1 (Section 2.3) is a well-known blocker of type I IFN induction. However, this block is not solely due to interactions with CPSF30 and inhibition of mRNA processing. Type I IFN induction is also blocked by NS1 through inhibition of the activation of the transcription factor IRF3, which controls IFN transcription (reviewed in [51]). This is a mechanistically separate effect that ultimately abolishes type I IFN transcription (reviewed in [51]). In addition, NS1 also blocks type I IFN signaling at downstream steps by interactions with double-stranded RNA, which prevents the activation of two anti-viral ISGs, protein kinase R (PKR) and 2′–5′ oligo A synthetase (OAS) (reviewed in [51]). The IFN-inhibiting strategy varies depending on the influenza A strain. Some variants of NS1 do not directly prevent IRF3 activation, for example Udorn NS1 and NS1s from other H3N2, H2N2 and some pre-2009 H1N1 strains, including A/AA/Huston/1945, A/Malaysia/1954, and A/USSR/46/1979 [55,67]. In these strains, interference with polyadenylation is the main mechanism of IFN inhibition [55,67]. In contrast, other pre-2009 H1N1 strains, including A/Texas/36/1991, and H5N1 strains after 1997 have both the IRF3 inhibition and the polyadenylation block functions [53,55,65], complicating the separation of these two modalities. There are also NS1 variants, like pre-1997 H5N1 isolates, the original pH1N1 isolates and PR8, that do not block polyadenylation at all, and only inhibit type I IFN by blocking IRF3 activation [52,53,55,65,98]. Many avian isolates of H6, H9 serotypes also have residues at NS1 aa 103 and 106 that suggest their NS1 cannot bind CPSF30 and interfere with polyadenylation [52,65]. One further complication to this analysis is that the CPSF30 binding capability of NS1 in isolation vs. in the context of the native virus may differ, and other viral proteins may facilitate NS1-CPSF30 binding [65,99]. Therefore, the use of precise mutations is needed to separate the role of the mRNA processing inhibition from other effects of NS1 on type I IFN induction.

One of the first pieces of evidence for the role of the polyadenylation block in type I IFN regulation is a report by Noah et al. demonstrating that mutations in the CPSF30 interaction site (aa 184–188, Table 2) reduce the ability of the Udorn strain to replicate in MDCK cells [67]. Moreover, naturally occurring mutations that reduce CPSF30 binding in the NS1 of seasonal H3N2 strains lead to higher cytokine induction and reduced virulence in mice [69,70]. Interestingly, adaptation to humans may select for CPSF30 binding. As mentioned in Section 2.3, amino acids F103 and M106 are key for NS1-CPSF30 interactions [61,62]. Between 1997 and 2004, these residues changed in H5N1 isolates (from L103 and I106 to F103 and M106), and this change may have contributed to increased virulence [65]. Clark et al. also recently reported that while NS1 from the original 2009 pH1N1 strains does not bind CPSF30, the NS1 from currently circulating pH1N1 strains has accumulated six mutations that together restore strong CPSF30 binding and host shutoff activity [68]. Indeed, this NS1 isoform is more efficient at reducing gene expression in reporter assays [68]. At least some of these changes appeared as early as the start of the 2009/2010 northern hemisphere season, almost immediately following introduction of the 2009 pH1N1 strain in the human population, and have all since gone to fixation [68]. These results suggest that there may be a selection for IFN inhibition through CPSF30 binding in the human host.

While restoring CPSF30 binding always restores host shutoff activity, it has variable effects on the virulence of the strain. Comparison of CPSF30-binding vs. non-binding isoforms of NS1 in H3N2, H5N1, H7N9 and canine H3N8 strains shows that CPSF30 binding promotes viral replication in cells and/or animal models, and increases virulence [64,65,66,69,70,100]. This is likely due to improved control of the production of innate immune and inflammatory cytokines. Conversely, in the case of pH1N1, restoration of CPSF30-binding via targeted mutations [52] or natural strain evolution [68] reduces morbidity and mortality in mice, perhaps because it reduces inflammation and immunopathology. Clark et al. compared recombinant strains with the original 2009 pH1N1 segments 1–7 and either segment 8 from the original strain or from currently circulating pH1N1 that show CPSF30 binding [68]. They found that NS1 variant from circulating pH1N1 strains was more effective in dampening induction of cytokines, like IFNβ, TNF, and CXCL10, and of ISGs like IFIT2 [68]. However, this increased ability to modulate host responses did not cause changes in viral titers in cell culture or mouse lung tissue [68]. Moreover, the recent NS1 variants actually reduced virulence in mouse infections [68]. Previously Hale et al. had observed the same phenotypes with targeted mutations of pH1N1 NS1 [52]. Clark et al. propose that the changes that have accumulated in NS1 since 2009 reflect better human adaption of the virus [68]. Similarly, the NS1 from equine H3N8 strains have evolved from original avian strains and have acquired mutations that abolish host shutoff activity (i.e. CPSF30 binding) [101]. Restoring CPSF30 binding makes the virus less fit in equine cells [101]. The strain-specific discrepancies likely arise from a combination of factors, including the biology of the host and the level of cytokines that the strain induces in the absence of the NS1 host shutoff activity. Moreover, given the multifunctional nature of NS1, different strains may have evolved an optimal combination of the different activities of this protein. Lastly, one cannot exclude that some of the mutations may also affect other NS1 functions, at least in some of the strains.

The discovery of PA-X (Section 2.1) and its classification as a host shutoff protein spurred many studies on the role of this novel protein on influenza A virulence and pathogenesis. Studies with viruses carrying mutations in the frameshifting site (PA(fs) viruses) have proved particularly valuable as they can be used to generate PA-X null (or at least low PA-X) viruses to test the function of this protein in vivo and in vitro. The in vivo studies with the PA(fs) viruses have provided extensive evidence that PA-X down-regulates innate immune and pro-inflammatory cytokines during infection, and thus acts as an immune modulator [12,20,21,102,103]. This role in innate immune evasion was shown with 1918 H1N1, pH1N1 and H5N1 strains, in which reduced PA-X expression resulted in increased induction of pro-inflammatory cytokines including TNF-alpha, IL-1, IL-6, IL-12α, IFN-α, IFN-β, and IFN-γ in cells as well as in mice and chickens [12,20,21,102,103]. H9N2 strains are currently outliers, with PA-X mutations reducing cytokine induction [104]. In addition to innate immune activation, PA-X also affects humoral adaptive immune responses. Mouse infection with a pH1N1 PA(fs) strain caused increased production of neutralizing antibodies [21]. While more work is needed to understand the mechanism behind this observation, this study provides evidence for the pervasive role of PA-X in modulation of the full organismal response to infection. In addition, this finding could have interesting implications for future influenza vaccine design [24]. 

The down-regulation of pro-inflammatory cytokine production, the consequent down-regulation of antiviral responses and the reduced recruitment of immune system cells should benefit the virus and promote replication and spread. Indeed, the increased host responses in strains lacking NS1 make the viruses less able to establish infection in cells and mice [49]. However, the analysis of PA(fs) viruses has revealed that down-regulation of host responses by influenza A virus can also have protective effects for the host. In fact, the original discovery of PA-X in the 1918 H1N1 influenza strain garnered attention in part because the phenotype of 1918 PA(fs) viruses is paradoxical [12]. While mutations in the frameshifting site increase host responses to infection, they do not compromise the ability of viruses to replicate in vivo, and in fact they result in higher mortality in infected mice and chicken in a 1918 chimeric strain [12] and other strain backgrounds, including pH1N1 and H5N1 [20,102,103]. In general, the histopathologic score and lung immune cell infiltrates in mice infected with PA-X-deficient viruses are increased [12,20,102,103]. These results indicate that stronger induction of these pro-inflammatory cytokines in the absence of PA-X can lead to increased lung immunopathology and subsequent increases in morbidity and mortality.

We have to note that there are discrepancies in the literature when it comes to the overall effect of PA-X in morbidity and mortality during infection. While some studies have reported no change in titer, increased immune activation and increased mortality [12], others have reported higher titers, increased immune activation and increased mortality [20,102,103,105], others reduced titers, increased immune activation and reduced mortality [21,106], and yet others reduced titers, reduced immune activation and reduced mortality [104]. Thus, while collectively these studies all argue for an important role of PA-X in host responses, they disagree on the specific effect of this protein. It is possible that the PA-X shutoff activity varies between different strains of influenza A virus, which could affect the overall pathogenicity of the strains. For example, Desmet et al. reported that PA-X variants from human adapted strains (A/New Caledonia/20/99 H1N1 and WSN) have lower activity than avian-origin PA-X (A/chicken/Nanchang/3-120/01 H3N2 and Cal pH1N1, which has a PA segment of avian origin) [18]. Of note, these experiments were carried out by expressing the PA mRNA and naturally obtaining PA-X from frameshifting. Therefore, it is possible that there may also be differences in frameshifting efficiency and thus PA-X production among strains, which could also affect the extent of host shutoff. Another possibility is that the ultimate phenotype of PA-X mutant strains is influenced by other characteristics of the strain background, including how immunoreactive the strain is in the first place [24]. For example, in strains that already induce strong immune activation losing PA-X may increase the inflammation to pathological levels, while in strains that induce poor innate immune responses the increase may boost clearance. Nonetheless, this explanation would not resolve all the conundrums in the field, since studies purportedly using very similar strains have also obtained opposite results. We note that there is currently no evidence for substantial differences in molecular function among PA-X variants that could give rise to the different effects on virulence, in contrast to what seen with NS1 [20,22].

An addition level of complexity is presented by the existence of two main variants of PA-X, with C-terminal X-ORFs of different lengths (41 aa vs. 61 aa). At present it is unclear how this difference impacts the immunomodulatory capacity of PA-X. A few studies have sought to explore effects of the X-ORF length on in vivo infections. This was achieved by inserting stop codons at aa 233 in strains that normally have a 252-aa PA-X (and thus shortening a 61-aa X-ORF to 41-aa) or by moving the natural stop codon from aa 233 to aa 253 in strains that normally have a 232-aa PA-X (and thus lengthening a 41-aa X-ORF) [33,107]. Gao et al. reported that strains with longer PA-X caused higher induction of many cytokines and greater mortality in the pH1N1, H5N1 and H9N2 backgrounds [33]. Lee et al. also reported similar changes in a study of pH1N1 [107]. Some of these results are at odds with those obtained with PA(fs) mutants in the corresponding strains, because both X-ORF shortening and the PA(fs) mutations are reported to cause less shutoff activity, but the virulence phenotypes are not always consistent [21,33,104,107]. Thus, while there appears to be some differences in the immunomodulatory effects between these two PA-X X-ORF variants, these differences remain to be resolved. A potential role of the X-ORF truncation could be adaptation to particular species. Shi et al. noted that the truncation to a 41-aa X-ORF appeared independently in two influenza A subtypes that infect dogs, suggesting this change may be of functional significance [17]. Moreover, Xu et al. suggested that shorter isoforms support better viral replication and immune evasion in swine cells and pigs [35]. In general, understanding the PA-X mechanism of action will provide a clearer picture of its role in influenza A virulence and pathogenicity, as well as better tools to resolve the role of the X-ORF variants. Also, the interpretation of the in vivo results assumes that the activity of PA-X is non-specific and that all cytokines and immune responses will be equally affected. It is presently unclear whether this assumption is correct. The connection between the contribution of PA-X to influenza A virulence and pathogenesis and its molecular specificity will undoubtedly be an interesting field to explore as we uncover the molecular mechanism of action of PA-X.

### 3.2. Alternative Functions of Host Shutoff

An alternative proposed role for host shutoff is to reduce competition between viral and host mRNAs for host translational machinery. A corollary of this model is that it could “erase” the natural host gene regulation of the cell to allow for the selective expression of genes that are beneficial for viral replication. This model, however, is very hard to prove, and has only been clearly described in the herpesviral vhs host shutoff [108]. In influenza A virus, testing such a model is also complicated by the multiple host shutoff mechanisms. For example, pH1N1 strains with NS1 that bind CPSF30 replicate to similar titers as the original strains, in which NS1 does not bind CPSF30 [68]. While this result suggests that NS1 host shutoff is not involved in replication *per se*, these viruses presumably still carry out host shutoff through PA-X and the RdRp. In the context of PR8, mutating the frameshifting site reduced plaque size and accumulation of a subset of viral proteins, without alterations in viral mRNA levels, which may support a role for PA-X in clearing ribosomes for viral translation [22]. However, mutations in PA-X do not generally reduce viral titers in MDCK cells [12,20], and in some cases promote replication in the human epithelial A549 cells and in mouse lung tissue [20]. Some studies have reported that in the absence of frameshifting, the levels of PA mRNA and/or protein are higher, and that there is increased polymerase activity in a replicon assay [20,105]. When examining the 61-aa vs. 41-aa of pH1N1 PA-X, Lee et al. also suggested that mutating PA-X could alter polymerase activity [107]. It is possible that these changes are simply a result of the lack of frameshifting, which could change the stability and translation of the PA mRNA. Alternatively, there could be an indirect effect of PA-X host shutoff on polymerase activity. In either case, these findings complicate the analysis of the PA(fs) mutant viruses.

A third possibility is that some of the decrease in host gene expression is an accidental consequence of viral processes. This idea was the basis for the original model of RNA destabilization as a by-product of cap snatching. While the PA-X protein has now been shown to be responsible for most of the RNA destabilization, reduced transcription could still occur as a by-product of cap snatching. As mentioned above, the unprotected 5′ ends of cellular Pol II transcripts generated by cap snatching could be the trigger for removal of Pol II from the body of genes, thus reducing transcription. Whether this is an intended purpose of the process, or whether influenza has evolved to take advantage of it remain open questions. Nonetheless, the overwhelming evidence described above indicates that at least for NS1 and PA-X, the host shutoff is a deliberate effect of the proteins, with important immunomodulatory functions in the context of in vivo infections.

## 4. How Are the Multiple Host Shutoff Mechanisms Integrated?

Influenza A virus has only eight gene segments but devotes a significant percentage of its coding capacity to proteins that directly or indirectly interfere with host gene expression. This “investment” raises the question of why such a small virus has evolved many different mechanisms of host shutoff and suggests that remodeling of host gene expression is critical for successful influenza A infection. One potential explanation for this phenomenon is the broad host range of influenza A viruses. Influenza A strains (unlike influenza B) infect not only humans but also many other species, including mallard ducks, chickens, seals, dogs, pigs, Owston civets, and horses [109]. In order to successfully establish infection in many different species, influenza A virus may need a wide range of mechanisms to overcome host responses. This may arise from different host biology, so that one mechanism may not prove as efficient in one species as it is in another. Alternatively, NS1 and PA-X may not work at all in some species without prior adaptation, because of differences in the cellular proteins that these viral factors interact with. Influenza A virus could thus make use of these different mechanisms to adapt as it moves through different hosts with different selective pressures.

Having multiple host shutoff proteins also means the virus needs to carefully adjust their immunomodulatory activities. The Takimoto lab first proposed that the host shutoff activities of NS1 and PA-X may be balanced, because in strains that lack NS1-based host shutoff, like Cal pH1N1, PA-X has stronger shutoff activity [18]. Subsequent studies from the Martinez-Sobrido and Topham labs have provided evidence that strains may indeed adjust the relative activity of NS1 and PA-X during evolution [23,110]. Nogales et al. found that the sequence of both NS1 and PA-X is different between the original 2009 pH1N1 isolates and the currently circulating ones [23,68]. In addition to the NS1 changes reported in Clark et al. and mentioned above (Table 2) [68], currently circulating pH1N1 strains also have four amino acid changes in PA-X, three of which are in the unique C-terminal domain (Table 1) [23]. Like the NS1 changes, these polymorphisms first appeared prior to the start of the 2011/2012 northern hemisphere season and have since gone to fixation. Interestingly, while the recent evolutionary changes in pH1N1 strains increase the host shutoff activity of NS1, they reduce the host shutoff activity of PA-X [23]. The authors suggest that current pH1N1 viruses have regained NS1-mediated host gene expression inhibition, and that there has been a subsequent reduction of PA-X activity to compensate, otherwise the virus would be too lethal or attenuated [23]. Similarly, a cold-adapted vaccine pH1N1 strain (A/California/4_NYICE_E3/2009 H1N1) with both active PA-X and CPSF30-binding NS1 is attenuated relative to the viruses with only one active host shutoff protein [110]. These results provide evidence that there may be a careful balance between NS1 and PA-X activity. Perhaps excessive host shutoff activity prevents optimal viral replication, as it may reduce the expression of host proteins needed for viral replication or cause cell death. It is also possible, as Khaperskyy and McCormick have speculated, that NS1 and PA-X work at different times during infection to contribute to optimal viral replication and virulence [111]. More mechanistic studies are needed to demonstrate how the balance and co-operation between NS1 and PA-X is achieved and to see if this effect is conserved across different influenza A serotypes and strains.

## 5. Conclusions

Studying the biology of influenza A virus is complicated by numerous factors including the multi-functional properties of its proteins, the rapidly changing genome and its multiple hosts. Extensive studies have shown that influenza A virus has a remarkable ability to reprogram host cell genome expression and have uncovered multiple mechanisms that can play a role in this process, but the data are still unclear with respect to their relative importance during in vivo infections. Also, it is possible that the multiple options in gene regulation confer flexibility, and thus a fitness advantage, as the virus moves through multiple different hosts. Surprisingly, evidence from the study of PA-X and PA-X-mutant viruses suggests that host shutoff could be protective, because it spares the host from lung immunopathology by reducing inflammation. This could have repercussions on human disease, as severe influenza disease is coupled to stronger inflammatory responses and not necessarily to higher viral replication. More work is needed to completely understand the molecular mechanisms of host shutoff in influenza A virus infection and its role in virulence and pathogenesis.

## Figures and Tables

**Table 1:** Specific residues that affect the host shutoff activity of PA-X and the RdRp. “Strong/weak activity” for PA-X and the RdRp subunits refers to naturally occurring variants with high/low levels of host shutoff activity. “na” in “weak activity” column is indicative of residues that were identified as important for host shutoff based on loss of activity when the residue is experimentally mutated.

Host Shutoff Protein	Residue	Strong Activity	Weak Activity	Available Information on Function	Reference
**PA-X**	2	E	na	Needs to be acetylated by NatB for full activity	[36]
	80	E	na	Residues required for nuclease activity of PA/PA-X and also shown experimentally to affect shutoff activity	[10,11,12,24,32]
	106	L	na		
	107	P	na		
	108	D	na		
	119	E	na		
	134	K	na		
	192–197 (aa 0-15 of X-ORF)	(varies depending on strain)	na	Required for nuclear localization and activity (WSN, Cal, PR8)	[19,22,29]
	195, 198, 199, 202, 203, 206	R/K	na	Mutation to A or E prevents nuclear import and activity (WSN, Cal, PR8)	[19,22,29]
	100	V	I, A	Changes that arose in pH1N1 and reduce PA-X activity (“weak” variants also found in WSN for 100 and 221)	[18,23]
	204	N	S		
	221	R	Q		
	229	L	S		
	57	R	Q	Amino acid differences responsible for higher shutoff activity of pH1N1 vs. WSN	[18]
	62	I	V		
	65	S	L		
	4	F	na	Important for PA-X shutoff activity, potentially by allowing nuclear import (WSN)	[32]
	9	F	na		
	27	D	na		
	39	C	na		
	123	T	na		
	124	R	na		
	125	R	na		
	24	Y	na	Important for PA-X shutoff activity presumably by structurally supporting nuclease site (WSN)	[32]
	45	C	na		
	87	A	na		
	94	I	na		
	120	I	na		
	163	L	na		
	171	I	na		
	27	D	N	Changes that increase shutoff activity of equine H3N8 PA-X in conjunction with lengthening of X-ORF isoform	[34]
	231	S	F		
**PA (RdRp)**	550	L	I	Required for RdRp to direct Pol II degradation	[48]
**PB2 (RdRp)**	504	V	I		

**Table 2:** Specific residues that affect the host shutoff activity of NS1. “Strong/weak activity” for NS1 variants refers to naturally occurring variants with strong/weak CPSF30 binding. “na” in “weak activity” column is indicative of residues that were identified as important for host shutoff based on loss of activity when the residue is experimentally mutated.

Host Shutoff Protein	Residue	Strong Activity	Weak Activity	Available Information on Function	Reference
**NS1**	103	F	L	F103/M106 confer CPSF30 binding in many strains (not sufficient in pH1N1, canine H3N8)	[62,65]
	106	M	I, V		
	144	L	na	Required for CPSF30 binding (Udorn, WSN)	[61]
	aa 184–188	GLEWN	na	Required for CPSF30 binding in vitro and in cells (Udorn); K186 (instead of E186) in canine H3N8 prevents CPSF30 binding	[62,63,66,67,73]
	aa 223–237	ARTARSKVRRDKMAD	na	Required for PABPN (PABII) binding (Udorn)	[63]
	55	K	E	Changes to “strong” restore strong CPSF30 binding in pH1N1 strains (with F103, M106); D189N also alters host shutoff activity in circulating H3N2 strains and 1918 H1N1; D125G appeared in mouse adaptation of H3N2 human strain	[52,68,70,74,75]
	90	I	L		
	108	K	R		
	123	V	I		
	125	D	E, G		
	131	E	K		
	189	D	G, N		
	205	S	N		
	64	I	T	Changes to “weak” in circulating H3N2 strains reduce CPSF30 binding and IFN antagonism; V194I also reduces CPSF30 binding and host shutoff activity in 1918 H1N1 NS1	[69,70]
	194	V	I		
	98	L	S	Mutations that abolish CPSF30 binding during mouse adaptation of H3N2 strain	[74,75]
	180	V	A		
	96	E	na	Mutation causes temperature sensitive mRNA cleavage phenotype (Udorn)	[72]
	aa 191–195	EALQR	deleted	Deletion in H5N1 NS1 reduces CPSF30 binding and IFN antagonism	[71]
